# Hypomethylation of VTRNA2-1 promoter predicts adverse outcomes in peripheral artery disease

**DOI:** 10.1186/s13148-026-02087-z

**Published:** 2026-02-27

**Authors:** Ming-Lung Tsai, Yi-Chun Huang, Ming-Yun Ho, Jih-Kai Yeh, Chun-Chi Chen, Chin-Yuan Chia, Wen-Lang Fan, I-Chang Hsieh, Chi-Neu Tsai, Chao-Yung Wang

**Affiliations:** 1 Division of Cardiology, New Taipei Municipal TuCheng Hospital, New Taipei City, Taiwan; 2 School of Medicine, College of Medicine, Chang Gung University, Taoyuan City, Taiwan; 3 College of Management, Chang Gung University, Taoyuan City, Taiwan; 4 Division of Cardiology, Linkou Medical Center, Chang Gung Memorial Hospital, 5 Fuxing Street, Guishan District, Taoyuan City, 33305 Taiwan; 5 Department of Medical Research, Kaohsiung Chang Gung Memorial Hospital, Kaohsiung, Taiwan; 6 Graduate Institute of Clinical Medical Sciences, Chang Gung University, Taoyuan, Taiwan; 7 Institute of Cellular and System Medicine, National Health Research Institutes, Zhunan, Taiwan; 8 College of Life Sciences and Medicine, National Tsing Hua University, Hsinchu, Taiwan

**Keywords:** Peripheral artery disease, Epigenetics, DNA methylation, VTRNA2-1, Biomarker, Cardiovascular outcomes, Risk stratification, Revascularization

## Abstract

**Background:**

Peripheral artery disease (PAD) is a progressive vascular condition with high risks of limb amputation and adverse clinical outcomes. Despite advancements in revascularization techniques, risk stratification remains limited, especially in predicting long-term limb-specific complications. Epigenetic markers, such as DNA methylation, have emerged as potential tools for improving prognostication in vascular disease.

**Methods:**

We prospectively enrolled 133 patients with symptomatic PAD confirmed by imaging and angiography, all of whom underwent endovascular or surgical revascularization. Peripheral blood samples were collected prior to intervention, and *VTRNA2-1* promoter methylation levels were quantified using bisulfite pyrosequencing. Hypomethylation was defined as methylation levels below 40%. Patients were followed for 12 months to monitor major adverse limb events (MALE), amputation, and renal outcomes. Methylation levels were compared to those of population-based controls from the Taiwan Biobank.

**Results:**

Among enrolled patients, 42.9% exhibited *VTRNA2-1* hypomethylation. Hypomethylation was independently associated with increased amputation risk (odds ratio [OR] 2.19, 95% confidence interval [CI] 1.09–4.42, *p* = 0.035). In patients without end-stage renal disease (ESRD), hypomethylation was associated with higher rates of MALE (OR 2.78; 95% CI, 1.11–6.97; *p* = 0.028). Additionally, methylation levels in PAD patients were significantly lower than those in population-based controls.

**Conclusion:**

*VTRNA2-1* hypomethylation is associated with increased risk of limb-related complications in PAD patients, especially among those without ESRD. These findings suggest that *VTRNA2-1* methylation status may serve as a promising biomarker for refining risk stratification in PAD. Further validation in external cohorts and mechanistic studies are warranted.

**Supplementary Information:**

The online version contains supplementary material available at 10.1186/s13148-026-02087-z.

## Introduction

Peripheral artery disease (PAD) represents a major cardiovascular challenge, affecting an estimated 236 million people worldwide [1]. PAD leads to chronic wounds, tissue necrosis, amputation, and death, with substantial healthcare burden. Despite established associations with traditional risk factors including diabetes, dyslipidemia, and altered waist-to-hip ratio, clinicians face significant challenges in predicting which patients will experience adverse outcomes following diagnosis and revascularization. [2–4] This unpredictability in disease progression suggests the involvement of underlying biological mechanisms beyond conventional risk factors.

Recent advances in epigenetics have revealed that early-life programming may significantly influence cardiovascular disease development [5–8]. The methylation status of genomic regions, particularly those sensitive to early environmental conditions, can create lasting signatures that affect disease susceptibility throughout life. Among these, the Vault RNA 2 − 1 (*VTRNA2-1*) differentially methylated region has emerged as a crucial epigenetic modifier that may bridge early-life experiences with adult disease risk [9, 10].

*VTRNA2-1* methylation status represents a crucial mechanistic link between developmental programming and cardiovascular pathology. Compelling evidence from studies on prenatal famine exposure demonstrates the sensitivity of DNA methylation patterns to early-life environmental influences, with these epigenetic modifications persisting into adulthood [5–7, 11]. The developmental plasticity of *VTRNA2-1* methylation exhibits remarkable specificity, as the effects on offspring metabolism and cardiovascular risk vary depending on the precise timing of exposure during gestation. Uniquely among human imprinted genes, *VTRNA2-1* methylation status is not determined by genetics, demonstrating exceptional resistance to environmental effects in adulthood while maintaining stable patterns across somatic tissues throughout life [12]. This stability, coupled with *VTRNA2-1*’s regulatory roles in critical pathways including insulin signaling, inflammation, and cellular senescence, provides a robust mechanistic foundation for its potential as a lifelong biomarker of cardiovascular risk [12–15]. Supporting this concept, the ‘Developmental Origins of Health and Disease’ hypothesis emphasizes how early-life exposures can induce lasting changes in metabolism and cardiovascular health through epigenetic modifications [16]. Twin studies have further validated the role of such epigenetic mechanisms in cardiovascular disease pathogenesis, allowing differentiation between genetic and environmental contributions to disease development [17, 18].

Evidence from related conditions strengthens the potential relevance of *VTRNA2-1* methylation in cardiovascular disease. Research has linked its methylation status to body mass index (BMI) and insulin sensitivity in early childhood [12]. Regarding cellular aging, Kim et al. demonstrated that *VTRNA2-1* methylation status and increased expression ameliorated cellular senescence in fibroblasts by inhibiting age-related biomarkers [13]. Additionally, in diabetic patients, hypomethylation of *VTRNA2-1* (< 40% methylation) was significantly associated with poor glycemic response to GLP-1 treatment [14]. These studies highlight *VTRNA2-1*’s impact on gene regulation and clinical outcomes through multiple pathways including insulin sensitivity, obesity, drug response, inflammatory responses, cellular proliferation, and apoptosis [12–15].

The potential application of *VTRNA2-1* methylation status in precision medicine for PAD represents an exciting frontier. While its role in cellular processes and metabolic regulation is increasingly recognized, its specific impact on cardiovascular outcomes, particularly in PAD, remains unexplored. The methylation status of the *VTRNA2-1* region is likely influenced by maternal environmental factors and affects multiple pathways related to cardiovascular event risk.

This study aims to bridge this critical knowledge gap by investigating the association between *VTRNA2-1* promoter methylation status and clinical outcomes in PAD patients requiring revascularization. By elucidating this relationship, we hope to identify new biomarkers for disease prognosis and potential targets for therapeutic intervention. Our findings could advance the integration of epigenetic profiling into clinical practice, potentially transforming risk assessment and treatment selection in PAD management.

## Method

### Study population

We enrolled patients with peripheral artery disease (PAD) who required revascularization therapy. Inclusion criteria comprised: (1) age ≥ 20 years; (2) written informed consent; and (3) PAD symptoms (Rutherford Classification Category 1–6) with confirmatory imaging (computed tomographic angiography, magnetic resonance angiography, or ultrasound) and subsequent angiographic demonstration of ≥ 50% stenosis necessitating angioplasty. Exclusion criteria were: (1) unwillingness to provide blood samples or participate in follow-up; (2) presence of severe comorbidities with life expectancy < 1 year; (3) decompensated heart failure; and (4) pregnancy or lactation. To date, this study is the first to investigate the association between *VTRNA2-1* promoter methylation levels and prognosis in patients with PAD. Therefore, no previously published studies are available that provide appropriate data for calculating the effect size required for sample size estimation.

The study protocol was approved by the Institutional Review Board of Chang Gung Medical Foundation (IRB No. 202000472A3; 202201980A3C501; 202301387A3; 202301900B0; 202300971A3). All participants provided written informed consent. For methylation status comparisons, we utilized de-identified control samples from the general population obtained through the Taiwan Biobank, a biological database of the Taiwanese population. These Biobank samples were used solely to demonstrate the background population distribution of methylation and were not included in outcome or regression analyses. Because detailed demographic and clinical data (e.g., age, sex, comorbidities, and medication use) were not available for matching, this comparison should be interpreted descriptively rather than inferentially. *VTRNA2-1* promoter hypomethylation was defined as methylation levels < 40%. This study was conducted in accordance with the principles outlined in the Declaration of Helsinki [19].

### Sample collection and processing

Blood samples were maintained on ice and processed within 30 min of collection. For leukocyte isolation, 10 mL of whole blood was combined with red blood cell lysis buffer and incubated at room temperature for 6 min, followed by centrifugation (4000 g, 4 °C, 2 min). The leukocyte pellet underwent a second brief lysis step (30 s) with red blood cell lysis buffer and was centrifuged under identical conditions.

The resulting leukocyte pellets were equally divided and processed for different analyses. For protein analysis, 60 µL cell lysis buffer (Cell Signaling) containing protease inhibitors (Roche) was added to one aliquot. For RNA analysis, 50 µL RNAlater (Ambion) was added to another aliquot. DNA was isolated from EDTA blood samples using DNeasy blood kit (Qiagen) per manufacturer’s protocol.

### DNA methylation analysis

CpG site-specific DNA methylation at *VTRNA2-1* was quantified using bisulfite pyrosequencing (QiagenPyroMark Q96 and Q24). The targeted region spans chr5:135,415,693–135,416,613 (GRCh37/hg19), encompassing four functionally relevant CpG sites, cg06536614 (chr5:135,416,381), chr5:135,416,388, cg26328633 (chr5:135,416,394), and cg25340688 (chr5:135,416,398) [14]. The assay was validated using standard controls of 100% methylated, 0% unmethylated, and unmodified human genomic DNA (EpiTect Control DNA and Control DNA Set, Qiagen). Genomic DNA was extracted from peripheral blood leukocytes, ensuring minimal erythrocyte contamination, and 1 µg of DNA was subjected to bisulfite conversion using the EpiTect Fast Bisulfite Conversion Kit (Qiagen), following the manufacturer’s protocol.

For PCR amplification, 10–20 ng of bisulfite-converted DNA was used. *VTRNA2-1* forward and reverse primers, designed using PyroMark Assay design software (Qiagen), are detailed in the supplemental Table 1. PCR was conducted on a PCR Workstation (Applied Biosystems) using 0.22 μm-filtered nuclease-free water (Invitrogen). All pipettes were calibrated and cleaned prior to PCR setup.

For pyrosequencing preparation, biotinylated PCR product (20 µL) was combined with Streptavidin Sepharose (1 µL; GE Healthcare) and binding buffer (40 µL; Qiagen), followed by 10-minute shaking for immobilization. The immobilized products were purified to single-stranded DNA using a vacuum workstation (Qiagen) per manufacturer’s protocol. The purified products were mixed with sequencing primer (0.3 µM; detailed in the supplemental Table 1) in annealing buffer (Qiagen), heated to 80 °C for 5 min, then cooled to room temperature for primer annealing. Pyrosequencing was performed on PyroMark Q96 or Q24 (Qiagen) with appropriate plates, enzymes, substrates, and nucleotides.

Methylation percentages were calculated as the average across the four targeted CpG sites, and hypomethylation was defined as < 40%, based on prior literature and ROC-optimized cutoffs validated in our previous studies [14]. Additionally, aliquots of whole blood were preserved for potential future RNA and protein expression studies related to *VTRNA2-1* functional activity.

### Follow-up and outcomes

All patients were followed for one year after enrollment. The primary outcome was major adverse limb events (MALE), comprising mortality, amputation, myocardial infarction, revascularization for PAD, and ischemic stroke. Secondary outcomes included amputation levels (categorized as above-knee, below-knee, foot, or no amputation) and major bleeding events. A prespecified subgroup analysis was conducted in patients without end-stage renal disease (ESRD) at baseline, which included assessment of progression to ESRD during follow-up.

### Statistical analysis

Comparisons of baseline characteristics and laboratory data between hypomethylation and non-hypomethylation groups were performed using Fisher’s exact test for categorical variables and independent t-tests for continuous variables. The Mann-Whitney U test was used to compare the distribution of *VTRNA2-1* promoter methylation levels between PAD patients and non-PAD controls, given the non-parametric nature of methylation data. The relationship between *VTRNA2-1* promoter methylation status and clinical outcomes was assessed using Fisher’s exact test for categorical comparisons. Additionally, we performed logistic regression analysis to calculate odds ratios (ORs) with 95% confidence intervals (CIs) for hypomethylation-associated risks. Subgroup analyses were conducted for age, sex, body mass index (BMI), and end-stage renal disease (ESRD), and interaction terms were tested to evaluate effect modification within these groups. A separate analysis was conducted for patients without end-stage renal disease at enrollment to evaluate the specific effects of *VTRNA2-1* promoter hypomethylation on clinical outcomes. Statistical significance was defined as *p* < 0.05. All analyses were performed using SPSS version 22.0 (IBM Corp, Armonk, NY).

## Result

In this prospective study, we enrolled 133 patients with PAD requiring revascularization therapy. Based on *VTRNA2-1* promoter methylation status, participants were stratified into two groups: 57 patients (42.9%) exhibited hypomethylation and 76 patients (57.1%) demonstrated non-hypomethylation patterns. Comprehensive baseline clinical characteristics were comparable between the two groups. These included demographic parameters (age, gender, body mass index), cardiovascular risk factors (smoking status, hypertension, diabetes mellitus), comorbidities (chronic kidney disease, end-stage renal disease, dyslipidemia, gout, chronic obstructive pulmonary disease, heart failure), and previous cardiovascular events (ischemic stroke, myocardial infarction, prior percutaneous coronary intervention, coronary artery bypass grafting). Additionally, laboratory parameters and Rutherford classification were similarly distributed between groups, encompassing complete blood count indices (hemoglobin, platelet count), renal function markers (blood urea nitrogen, serum creatinine), electrolytes (sodium, potassium), metabolic parameters (uric acid, alanine aminotransferase), and lipid profile components (HDL-cholesterol, LDL-cholesterol, total cholesterol, triglycerides), with no statistically significant differences observed (Table 1).

**Table 1:** Characteristics of patients with or without DNA hypomethylation

Variable	Hypomethylation (*n* = 57)	Non-Hypomethylation (*n* = 76)	*P*
Age	67.05 ± 11.59	67.75 ± 12.87	0.747
Male	32 (58.6%)	46 (60.5%)	0.611
Body mass index, kg/m^2^	24.17 ± 4.10	24.66 ± 4.10	0.509
Smoke	19 (33.3%)	29 (38.2%)	0.566
Comorbidity			
Hypertension	42 (73.7%)	62 (81.6%)	0.275
Diabetes mellitus	47 (82.5%)	62 (81.6%)	0.896
Chronic kidney disease	33 (57.9%)	45 (59.2%)	0.879
End stage renal disease	21 (36.8%)	33 (43.4%)	0.445
Dyslipidemia	15 (26.3%)	23 (30.3%)	0.618
Gout	5 (8.8%)	6 (7.9%)	1.000
COPD	2 (3.5%)	4 (5.3%)	0.698
Ischemic stroke	7 (12.3%)	17 (22.4%)	0.134
Heart failure	18 (31.6%)	14 (18.4%)	0.079
Myocardial infarction	6 (10.5%)	9 (11.8%)	0.812
PCI	14 (24.6%)	9 (11.8%)	0.055
CABG	4 (7%)	1 (1.3%)	0.164
Atrial fibrillation	9 (15.8%)	10 (13.2%)	0.668
Gastrointestinal bleeding	12 (21.1%)	11 (14.5%)	0.321
Malignancy	3 (5.3%)	5 (6.6%)	0.752
Laboratory data			
Hemoglobin, g/dL	10.54 ± 2.26	11.20 ± 2.30	0.104
Platelet,	259.35 ± 106.04	275.95 ± 147.71	0.476
Blood urea nitrogen ^#^	23.34 ± 12.20	26.07 ± 13.73	0.361
Serum creatinine, mg/dL ^#^	1.05 ± 0.46	1.28 ± 0.84	0.118
Sodium (Na), mEq/L	137.79 ± 6.41	136.51 ± 2.98	0.168
Potassium (K), mEq/L	4.09 ± 0.76	4.29 ± 0.77	0.140
Uric acid, mg/dL	5.35 ± 1.52	7.51 ± 9.89	0.218
ALT, U/L	19.48 ± 15.11	20.06 ± 14.77	0.834
HDL-C, mg/dL	39.74 ± 14.07	39.61 ± 14.98	0.965
LDL-C, mg/dL	86.42 ± 40.95	74.99 ± 40.86	0.167
Total cholesterol, mg/dL	153.36 ± 44.54	150.59 ± 42.23	0.743
Total glyceride, mg/dL	139.55 ± 91.22	146.83 ± 98.02	0.702
Rutherford category			0.852
6	3 (5.4%)	3 (3.9%)	
5	39 (69.6%)	54 (71.1%)	
4	13 (23.2%)	16 (21.1%)	
3	0 (0%)	3 (3.9%)	
2	1 (1.8%)	0 (0%)	

ALT, alanine aminotransferase; CABG, coronary artery bypass graft; COPD, chronic obstructive pulmonary disease; HDL-C, High-density lipoprotein; LDL-C, Low-density lipoprotein; PCI, percutaneous coronary intervention

^#^Not including patients on dialysis

Data are presented as frequencies (percentages) or means ± standard deviations

### VTRNA2-1 methylation status in PAD

**Fig. 1:**
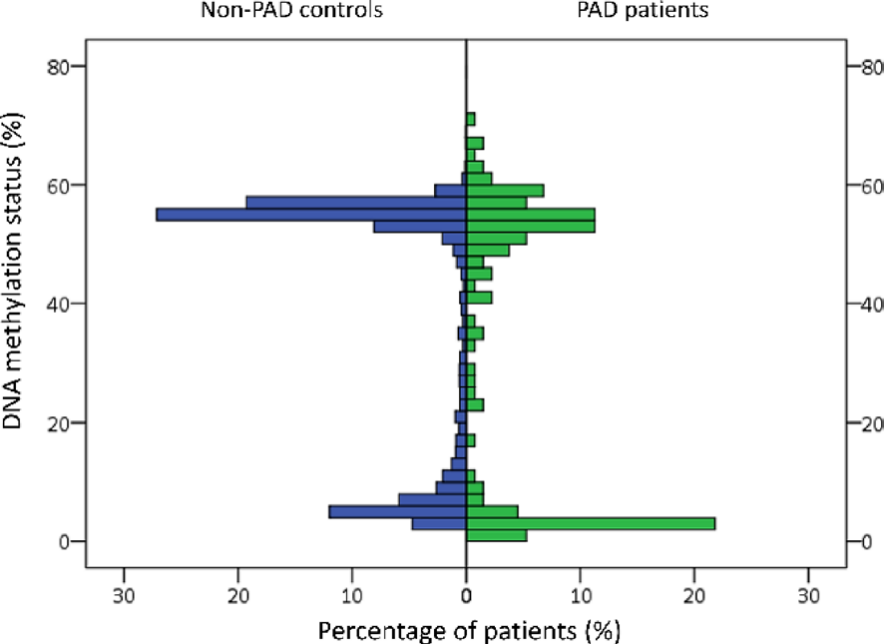
Distribution of *VTRNA2-1* promoter methylation status in PAD patients and non-PAD controls. The mirror histogram shows the distinct methylation patterns between PAD patients (right, green, *n* = 133) and non-PAD controls (left, blue, *n* = 2469). PAD patients demonstrated significantly different methylation patterns compared to controls (Mann-Whitney U tests, *P* < 0.001). PAD, peripheral artery disease

To determine whether PAD patients exhibit distinct *VTRNA2-1* methylation patterns, we utilized normal population control samples from the Taiwan Biobank for comparison. Analysis revealed that PAD patients demonstrated a significantly higher prevalence of *VTRNA2-1* promoter hypomethylation compared to the general Taiwanese population (*p* < 0.001, Fig. 1). The complete distribution patterns of *VTRNA2-1* promoter methylation status in both PAD patients and controls are presented in Supplementary Fig. 1A and B. This distinct methylation profile suggests that *VTRNA2-1* promoter hypomethylation may be a characteristic epigenetic feature of the PAD patient population.

### Clinical outcomes

Analysis of primary and secondary endpoints revealed distinctive patterns associated with *VTRNA2-1* methylation status (Table 2). The primary endpoint of major adverse limb events (MALE) demonstrated a trend toward higher occurrence in the hypomethylation group compared to the non-hypomethylation group (71.9% versus 57.9%; OR 1.86; 95% CI 0.89–3.89; *p* = 0.095). More striking was the significantly elevated risk of amputation in the hypomethylation group (56.1% versus 36.8%; OR 2.19; 95% CI 1.09–4.42; *p* = 0.035), while other outcomes including mortality, myocardial infarction, revascularization, and ischemic stroke showed no significant between-group differences.

**Table 2:** Follow-up outcomes

Variable	Hypomethylation (*n* = 57)	Non-Hypomethylation (*n* = 76)	OR (95% CI)	*P*
*Primary outcomes*				
MALE†	41 (71.9%)	44 (57.9%)	1.86 (0.89–3.89)	0.095
Any mortality	3 (5.3%)	7 (9.2%)	0.55 (0.14–2.22)	0.515
Any amputation	32 (56.1%)	28 (36.8%)	2.19 (1.09–4.42)	**0.035**
Myocardial infarction	1 (1.8%)	3 (3.9%)	0.44 (0.04–4.29)	0.635
Revascularization	16 (28.1%)	17 (22.4%)	1.35 (0.61–2.99)	0.451
Ischemic stroke	0 (0%)	2 (2.6%)	–	0.506
Other outcomes				
Amputation levels			–	**0.036**
AK	3 (5.3%)	1 (1.3%)		0.187
BK	4 (7.0%)	6 (7.9%)		0.849
Foot	25 (43.9%)	21 (27.6%)		0.051
Non	25 (43.9%)	48 (63.2%)		**0.027**
Any major bleeding	0 (0%)	2 (2.6%)	–	0.506

AK, above-knee amputation; BK, below-knee amputation; MALE, major adverse limb events

†Including any of the following: any mortality, any amputation, myocardial infarction, revascularization, or ischemic stroke

Data are presented as frequencies (percentages)

Statistical significance was defined as p < 0.05 and highlighted in bold

**Fig. 2:**
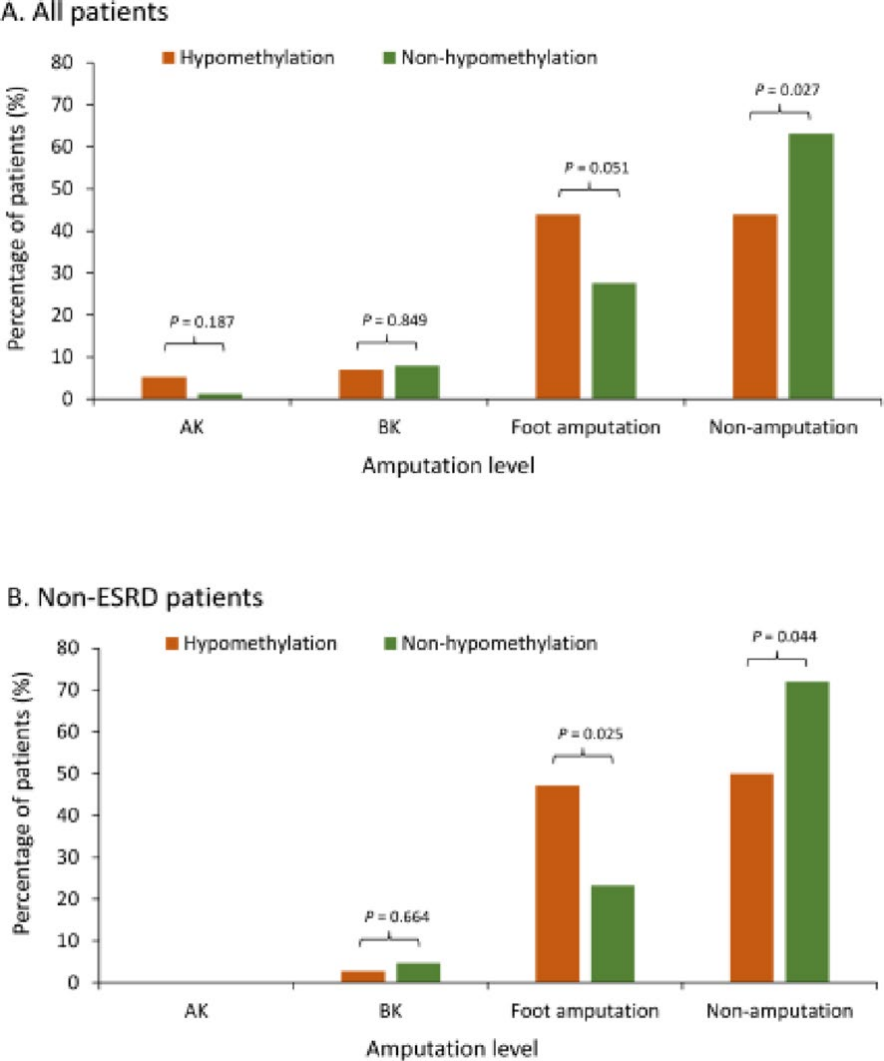
Amputation outcomes stratified by *VTRNA2-1* methylation status. **A** Amputation outcomes in all PAD patients (*n* = 113). Patients were stratified into hypomethylation (orange) and non-hypomethylation (green) groups. The number of patients in each category was as follows: above-knee (AK) amputation: *n* = 3 (hypomethylation), *n* = 1 (non-hypomethylation); below-knee (BK) amputation: *n* = 4 and *n* = 6; foot amputation: *n* = 25 and *n* = 21; non-amputation: *n* = 25 and *n* = 48.** B** Amputation outcomes in non-ESRD PAD patients (*n* = 79). The number of patients in each category was: above-knee (AK) amputation: *n* = 0 (both groups); below-knee (BK) amputation: *n* = 1 (hypomethylation), *n* = 2 (non-hypomethylation); foot amputation: *n* = 17 and *n* = 10; non-amputation: *n* = 18 and *n* = 31. Statistical analysis was performed using Fisher’s exact test. AK, above-knee amputation; BK, below-knee amputation; PAD, peripheral artery disease

**Fig. 3:**
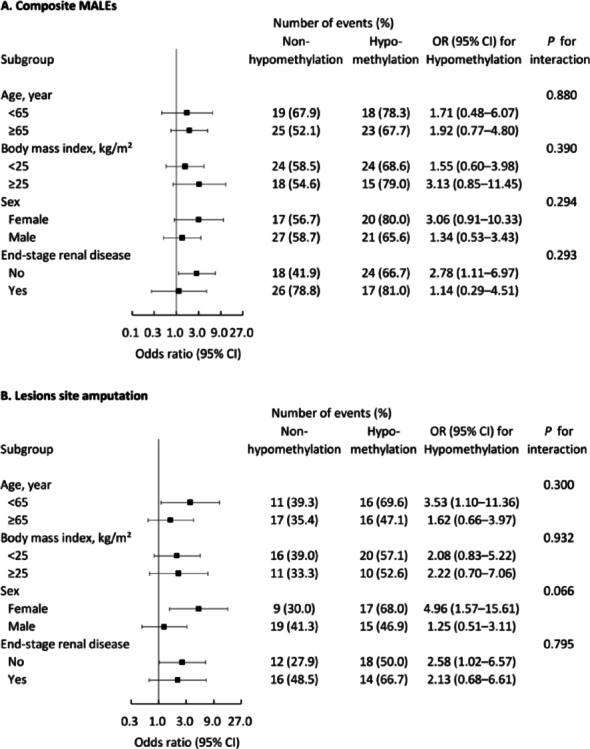
Subgroup analyses of clinical outcomes based on *VTRNA2-1* methylation status. **A** Major adverse limb events across patient subgroups (*n* = 85),** B** Lesion site amputation events across patient subgroups (*n* = 60). Statistical analysis was performed using Fisher’s exact test and logistic regression. MALE, major adverse limb events

Detailed analysis of amputation patterns revealed that hypomethylation was associated with increased rates of both above-knee and foot amputations (*p* = 0.035; Table 2; Fig. 2A). Specifically, patients with hypomethylation showed a trend toward more frequent foot amputations (43.9% versus 27.6%; *p* = 0.051). Conversely, patients in the non-hypomethylation group were more likely to avoid amputation during follow-up (63.2% versus 43.9%; *p* = 0.027). Subgroup analyses (Fig. 3A and B) showed consistent results across different levels of these subgroup variables (all* p* values for interaction > 0.05).

### Outcomes in non-ESRD patients

The impact of *VTRNA2-1* promoter hypomethylation was particularly pronounced in patients without end-stage renal disease (ESRD) at enrollment (Table 3; Fig. 2B). In this subgroup, hypomethylation was associated with significantly higher risks of both MALE (66.7% versus 41.9%; OR 2.78; 95% CI 1.11–6.97; *p* = 0.028) and amputation (50% versus 27.9%; OR 2.58; 95% CI 1.02–6.57; *p* = 0.044). Furthermore, these patients exhibited a significantly increased risk of progression to ESRD requiring dialysis (11.1% versus 0%; *p* = 0.039).

**Table 3:** Outcomes for patients without ESRD

Variable	Hypomethylation (*n* = 36)	Non-hypomethylation (*n* = 43)	OR (95% CI)	*P*
*Primary outcomes*				
MALE†	24 (66.7%)	18 (41.9%)	2.78 (1.11–6.97)	**0.028**
Any mortality	0 (0%)	2 (4.7%)	–	0.498
Any amputation	18 (50%)	12 (27.9%)	2.58 (1.02–6.57)	**0.044**
Myocardial infarction	0 (0%)	1 (2.3%)	–	1.000
Revascularization	10 (27.8%)	7 (16.3%)	1.98 (0.67–5.88)	0.215
Ischemic stroke	0 (0%)	1 (2.3%)	–	1.000
*Other outcomes*				
Amputation levels			–	0.065
AK	0 (0%)	0 (0%)		–
BK	1 (2.8%)	2 (4.7%)		0.664
Foot	17 (47.2%)	10 (23.3%)		**0.025**
Non	18 (50.0%)	31 (72%)		**0.044**
Any major bleeding	0 (0%)	1 (2.3%)	–	1.000
ESRD	4 (11.1%)	0 (0%)	–	**0.039**

AK, above-knee amputation; BK, below-knee amputation; MALE, major adverse limb events

†Including any of the following: any mortality, any amputation, myocardial infarction, revascularization, or ischemic stroke

Data are presented as frequencies (percentages)

Statistical significance was defined as p < 0.05 and highlighted in bold

## Discussion

To our knowledge, this is the first study to demonstrate that *VTRNA2-1* promoter methylation status is associated with clinical outcomes in peripheral artery disease, revealing a novel epigenetic dimension of cardiovascular risk. Our findings establish three key discoveries with immediate clinical relevance. First, PAD patients exhibit a distinct methylation signature compared to the general population (*P* < 0.001), suggesting a fundamental epigenetic basis for disease susceptibility. Second, *VTRNA2-1* promoter hypomethylation independently predicts adverse outcomes, most notably a more than twofold increased risk of amputation (OR 2.19, 95% CI 1.09–4.42, *p* = 0.035). Third, in patients without established end-stage renal disease, hypomethylation shows prognostic value for both major adverse limb events (OR 2.78) and progression to ESRD, identifying a high-risk subgroup that may benefit from more aggressive intervention.

These findings address a critical unmet need in PAD management: the ability to identify high-risk patients before clinical deterioration becomes evident. The predictive value of *VTRNA2-1* methylation status remains robust even after adjusting for traditional risk factors, suggesting its utility as an independent risk stratification tool. The strength and consistency of these associations, coupled with the stability of methylation patterns, positions *VTRNA2-1* methylation status as a promising biomarker for clinical implementation. While previous cardiovascular risk markers often fluctuate with disease state, methylation patterns remain stable, potentially offering a more reliable prognostic tool [20]. These findings open new avenues for precision medicine in PAD, where epigenetic profiling could guide both the timing and aggressiveness of interventions.

Subgroup analysis revealed particularly compelling findings in patients without established ESRD at enrollment, where *VTRNA2-1* promoter hypomethylation demonstrated enhanced predictive power. In this critical subgroup, hypomethylation was associated with a 2.78-fold increased risk of major adverse limb events (95% CI 1.11–6.97, *p* = 0.024) and a 2.58-fold higher amputation risk (95% CI 1.02–6.57, *p* = 0.044). Most strikingly, 11.1% of hypomethylated patients without baseline ESRD progressed to require dialysis during follow-up, compared to none in the normal methylation group (*p* = 0.039). These findings suggest that *VTRNA2-1* methylation status may identify a vulnerable patient subset before clinical deterioration becomes apparent. The enhanced predictive value in non-ESRD patients offers unique clinical opportunities. While ESRD itself profoundly affects peripheral vasculature, potentially masking other risk factors, *VTRNA2-1* hypomethylation appears most informative in earlier disease stages where intervention might yield the greatest benefit. This timing advantage is particularly relevant for clinical decision-making, as it could guide both the intensity and frequency of monitoring, as well as the threshold for aggressive intervention. The ability to predict both limb events and renal deterioration suggests that *VTRNA2-1* methylation status captures fundamental aspects of vascular health that transcend traditional risk stratification.

The robust clinical associations observed with *VTRNA2-1* methylation status may be understood through a unifying mechanistic framework centered on vascular repair and inflammation. Our findings suggest that hypomethylation of its promoter region creates a distinct molecular environment that promotes adverse vascular remodeling through multiple interconnected pathways. Central to this framework is the interaction between *VTRNA2-1* and the TGF-β pathway. Hypomethylation leads to increased *VTRNA2-1* expression, which is associated with elevated TGF-β1 levels [21, 22]. This relationship appears bidirectional, with TGF-β1 further influencing *VTRNA2-1* expression, potentially creating a feed-forward loop [23]. In the context of PAD, this interaction becomes particularly relevant during vascular repair, where TGF-β1 orchestrates the balance between adaptive and maladaptive responses. Our observation of increased amputation rates in hypomethylated patients aligns with experimental evidence showing that excessive TGF-β1 signaling promotes pathological fibrosis and impairs tissue healing.

Recent evidence further supports the downstream functional relevance of this locus. A large multi-cohort meta-analysis demonstrated that genetically and epigenetically predicted upregulation of *VTRNA2-1* is associated with adverse cardiometabolic traits, including higher diastolic blood pressure, lower HDL cholesterol, and increased risks of type 2 diabetes, stroke, and mortality [24]. These findings align with the biological model in which promoter hypomethylation at the *VTRNA2-1* locus leads to derepression of RNA transcription, thereby promoting proinflammatory and maladaptive vascular responses. Although our study did not directly measure *VTRNA2-1* RNA expression, the observed association between *VTRNA2-1* hypomethylation and adverse PAD outcomes may reflect this upstream epigenetic activation mechanism.

A second key mechanism involves *VTRNA2-1*’s regulation of the microRNA network through its interaction with Dicer [23]. This interaction affects multiple miRNAs critical for vascular homeostasis, including miR-21 (inflammation resolution), miR-126 (endothelial function), and miR-143/145 (smooth muscle phenotype) [25–29]. The unified effect of disturbing these pathways - impaired angiogenesis, enhanced inflammation, and dysregulated smooth muscle cell behavior - provides a molecular basis for the adverse outcomes observed in hypomethylated patients. Particularly relevant is the impact on miR-126, which regulates VEGF signaling and endothelial survival, directly linking to the poor outcomes in tissue healing and limb salvage [26].

These molecular mechanisms converge to create a “perfect storm” in PAD patients, where hypomethylation-induced dysregulation of both TGF-β and miRNA pathways compromises vascular repair capacity while promoting maladaptive remodeling. This model explains not only the increased risk of limb events but also the progression to ESRD, as similar pathways govern renal vascular health. The stability of these methylation patterns helps explain why their predictive value remains robust even after adjusting for traditional risk factors, as they reflect fundamental biological vulnerabilities that may not be captured by clinical parameters alone.

The profound impact of *VTRNA2-1* methylation status on cardiovascular outcomes raises a fundamental question about the origins of these epigenetic patterns. Our findings gain particular significance in light of emerging evidence that *VTRNA2-1* methylation is established during early development and persists into adulthood [6, 8]. This temporal stability distinguishes *VTRNA2-1* from other cardiovascular biomarkers that fluctuate with disease state, suggesting its role as both a marker of early-life programming and a determinant of adult disease susceptibility. However, whether *VTRNA2-1* methylation levels remain entirely stable in the setting of chronic vascular stress or systemic inflammation is unknown. Longitudinal studies with repeated methylation measurements and longer follow-up durations will be essential to clarify the temporal dynamics of this locus and determine whether changes in methylation over time correlate with disease progression or therapeutic response.

Maternal environmental conditions during pregnancy appear to play a crucial role in establishing *VTRNA2-1* methylation patterns. The persistence of these patterns into adulthood suggests a mechanism by which early-life exposures can influence cardiovascular risk decades later. In PAD specifically, this developmental perspective offers new insights into disease heterogeneity - patients with identical traditional risk factors may experience dramatically different outcomes based on their epigenetically-determined vascular repair capacity. This concept fundamentally shifts our understanding of PAD from a purely acquired condition to one whose trajectory may be influenced by developmental programming.

The implications extend beyond individual patient care to population health strategies. The stable nature of *VTRNA2-1* methylation patterns suggests opportunities for early-life interventions that could modify lifelong cardiovascular risk. Moreover, understanding these developmental origins opens new avenues for identifying at-risk individuals long before clinical manifestations appear, potentially enabling preventive strategies that could alter the natural history of vascular disease.

Current risk assessment in PAD relies heavily on clinical parameters and anatomical findings, which may fail to capture underlying biological vulnerabilities. Integration of *VTRNA2-1* methylation status could transform this paradigm, allowing for biology-driven risk stratification. The identification of *VTRNA2-1* methylation status as a predictive marker opens several therapeutic and clinical avenues. First, this epigenetic signature could guide the timing and aggressiveness of revascularization, particularly in patients without ESRD where its predictive value is strongest. Patients with hypomethylation might benefit from more frequent surveillance, earlier consideration of revascularization, or aggressive risk factor modification. Second, the mechanistic link to TGF-β signaling suggests potential therapeutic targets - ongoing trials of TGF-β pathway modulators might be particularly relevant for patients with *VTRNA2-1* hypomethylation. Third, the stability of methylation patterns makes *VTRNA2-1* an attractive biomarker for clinical implementation, potentially transforming risk stratification in vascular and renal diseases.

From a translational perspective, assessment of *VTRNA2-1* promoter methylation may complement current anatomical and hemodynamic assessments by identifying patients biologically predisposed to poor vascular repair or renal deterioration. In clinical practice, this information could help guide the intensity of post-revascularization surveillance, the timing of follow-up imaging, and the threshold for reintervention or systemic therapy optimization. However, before routine adoption, these applications will require validation in larger, prospective, and multicenter studies to establish reproducibility, standardization, and clinical cost-effectiveness. Incorporating epigenetic biomarkers such as *VTRNA2-1* methylation into clinical algorithms may eventually complement existing anatomic or physiologic scoring systems, contributing to a more comprehensive assessment of vascular risk.

### Limitations

Several limitations of this first translational investigation of *VTRNA2-1* methylation in PAD should be acknowledged when interpreting our findings. First, the non-randomized, observational design may introduce residual confounding, despite rigorous adjustment for established cardiovascular risk factors. As with all observational studies, causality cannot be definitively established, although our conclusions are strengthened by biological plausibility, temporality, and consistency across multiple outcomes. Second, the study was conducted at a single tertiary referral center and a regional hospital with a relatively modest sample size (*n* = 133), focused on a high-risk cohort undergoing revascularization. While this enhances clinical relevance for advanced PAD, the findings may not be generalizable to patients with milder disease or those managed conservatively. Furthermore, the limited sample size may restrict the detection of subtle subgroup interactions, although the statistical robustness observed in key comparisons (particularly in non-ESRD patients) supports the validity of the primary results. Third, although methylation analysis was performed in peripheral leukocytes, rather than vascular tissue, prior literature supports the use of circulating epigenetic biomarkers as systemic proxies for vascular health. Nevertheless, the tissue specificity of the VTRNA2-1 signal warrants further validation, particularly given the complex cellular heterogeneity of whole blood. Fourth, the bimodal distribution of *VTRNA2-1* methylation observed in our cohort may reflect underlying interindividual epigenetic variation but also highlights the need for independent validation of the threshold used. Population-specific or ancestry-related differences could influence the distribution and prognostic implications of this biomarker. Future multicenter studies across different countries and ethnic backgrounds are required to verify these findings and refine population-specific, clinically applicable cut-off values. Fifth, the lack of an independent external validation cohort limits the immediate generalizability of our findings. Larger multicenter studies are also needed to replicate these findings and validate population-specific thresholds for clinical application. Lastly, the one-year follow-up period, while offering actionable short-term outcomes, does not capture the full spectrum of long-term vascular and renal events. Extended follow-up is ongoing to better understand the durability of *VTRNA2-1* methylation as a prognostic marker.

Despite these limitations, our findings provide a compelling rationale for further research. Future investigations should focus on: (1) multicenter validation in racially and geographically diverse populations; (2) extended follow-up beyond one year; (3) functional studies to elucidate causal mechanisms linking *VTRNA2-1* to vascular pathology; and (4) evaluation of this epigenetic biomarker in earlier PAD stages and other vascular beds. These efforts will clarify the clinical utility of *VTRNA2-1* methylation and may enable its integration into personalized risk assessment strategies. They may also provide a mechanistic bridge between early-life epigenetic programming and later-life vascular outcomes, offering opportunities for both preventive and therapeutic innovation.

## Conclusion

The methylation status of the *VTRNA2-1* promoter represents a novel epigenetic determinant of outcomes in PAD, with hypomethylation predicting increased risks of amputation and renal failure progression. These findings establish a molecular link between developmental programming and vascular disease, while offering immediate clinical utility for risk stratification. By integrating *VTRNA2-1* methylation status into clinical assessment, we may better identify high-risk patients who could benefit from more aggressive intervention. This epigenetic signature opens new avenues for precision medicine in cardiovascular disease management.

## Supplementary Information

Below is the link to the electronic supplementary material.

Supplementary Material 1.

## Data Availability

The article and Supplementary Material include the original contributions presented in this study. Further inquiries can be directed to the corresponding author.
