# Supplementary material for: Hypomethylation of VTRNA2-1 promoter predicts adverse outcomes in peripheral artery disease
Source: Clin Epigenetics. 2026 Feb 27;18:55. doi: 10.1186/s13148-026-02087-z (PMC13049864; doi:10.1186/s13148-026-02087-z)
Supplement: Supplementary file 1 — Supplementary Material 1. [file 13148_2026_2087_MOESM1_ESM.docx]

**Supplement Table 1.** **Primers used for bisulfite sequencing primers (BSP), pyrosequencing reaction and centromeric CTCF (rs2346018) polymorphism**

| **Gene Name** | **Sense 5’-3’** | **Antisense 5’-3’** | **Size (bp)** | **Position**  **(GRCh37/hg19**  **Assembly )** |
| --- | --- | --- | --- | --- |
| VTRNA2-1 BSP | TTGAAATTTTTAAATTATAGAAGAGTGA | ATAAATAAATTTTACCCCCTTCCAC | 197 | chr5:135,416,303-135,416,499 |
| Pyrosequencing-VTRNA2-1 | AGAGGGAAGGGTTGTATGTGT | AAAAAAACTAAAAATCCCTCCAA (Biotin) | 113 | chr5:135,416,339-135,416,451 |
| Pyrosequencing-VTRNA2-1 Sequencing primer | TTTAGTTTTAGAGAGGTTTG |  |  | Chr5:135,416,358-135,416,380 |
| Centromeric CTCF site (rs2346018) | ATGGTGGTACCAGAGCAAGG | GAGAAAATGCCTGCGTCTCT | 178 | chr5:136,079,522-136,079,699 |

**
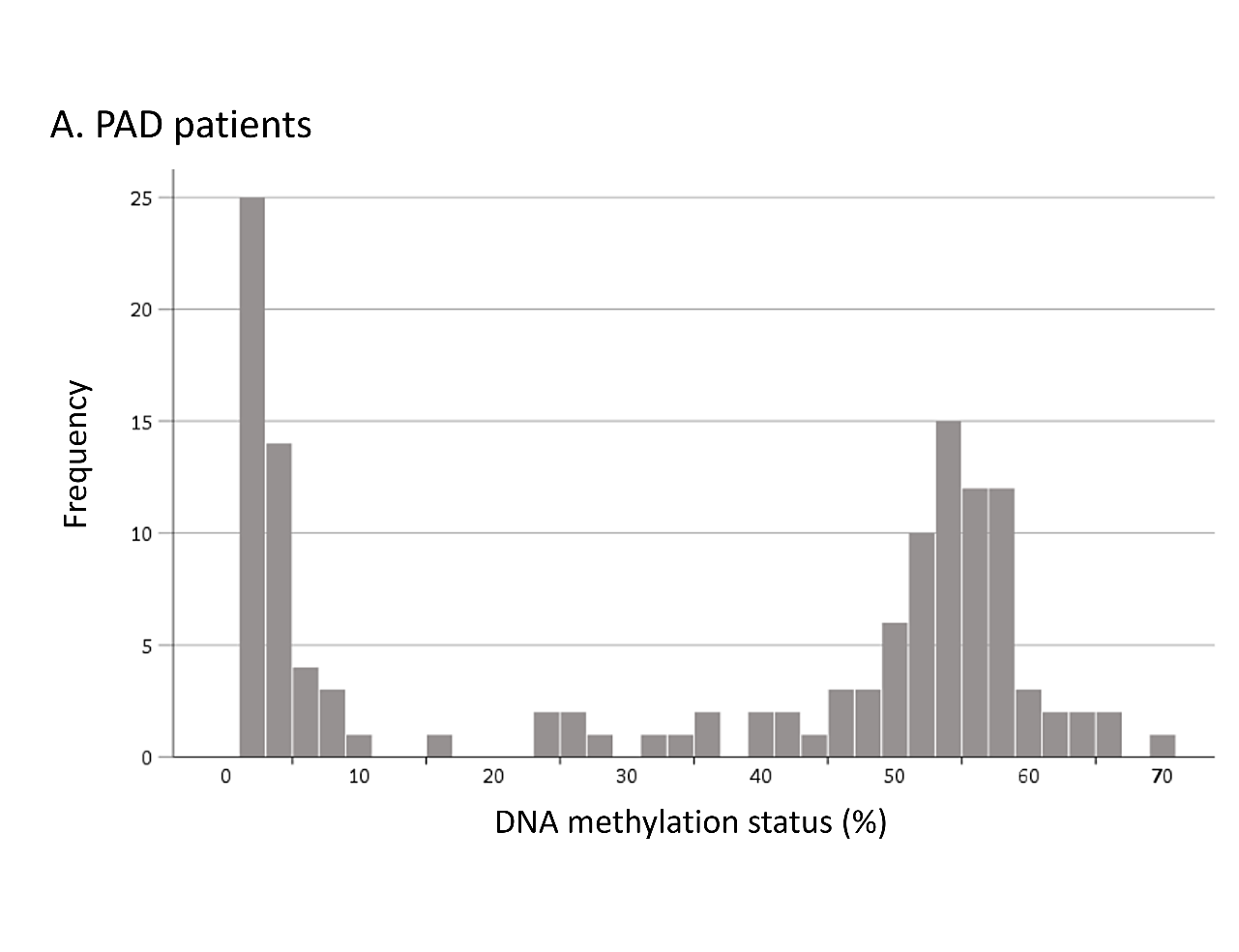

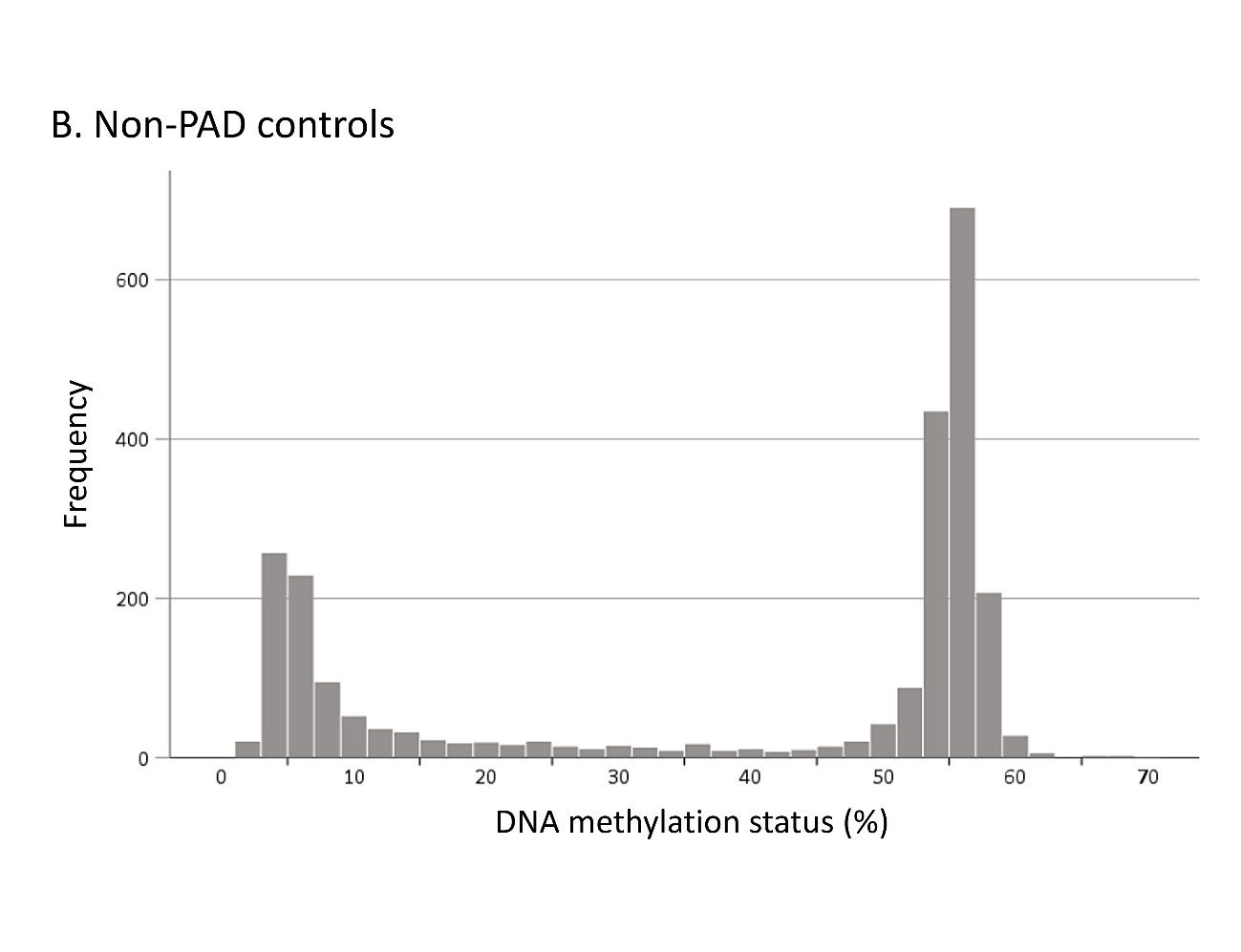
**

**Supplementary Figure 1.** **Detailed frequency distribution of VTRNA2-1 promoter methylation status.**

(A) Distribution of DNA methylation levels in PAD patients (*n* = 133), (B) Distribution of DNA methylation levels in non-PAD controls (*n* = 2,469. Statistical analysis was performed using descriptive statistics.
